# Surgical management of atrial-esophageal fistula as a complication of atrial fibrillation ablation

**DOI:** 10.1093/jscr/rjab497

**Published:** 2022-01-26

**Authors:** Amber Duda, Kevin Beers, Mohi Mitiek

**Affiliations:** Department of General Surgery, Mercy Health the Jewish Hospital, Cincinnati, OH, USA; Department of Cardiothoracic Surgery, Children’s Mercy Hospital, Kansas City, MO, USA; Department of Cardiothoracic Surgery, Children’s Mercy Hospital, Kansas City, MO, USA

## Abstract

Atrial-esophageal fistula (AEF) is a rare, but life-threatening complication of ablative treatments for atrial fibrillation. Although the incidence of this complication is low, the mortality is very high. There are many surgical approaches to this disease but we offer a novel technique to reduce the number of incisions used and provides central cannulation. It also allows for repair of both the esophagus and atrium and buttresses these repairs, which have both been shown to decrease morbidity and mortality. The technique has been successful in our three patients and can be considered as an approach to surgical management of AEF.

## INTRODUCTION

Atrial ablation is a common treatment for patients with atrial fibrillation. As the number of these procedures increase the complications rise [1]. The major complication rate following these procedures is 6% [2]. One of the most severe complications is an atrial-esophageal fistula (AEF). Fistula formation is thought to occur secondary to intraluminal thermal injury.

The incidence of this complication is between 0.03 and 1.5% [3]. The mortality following this complication ranges from 40 to 80% [3]. Surgical intervention is the recommended treatment, but successful intervention is limited. Clinical suspicion should remain high in patients status post ablative procedures who present with complaints including fever, hematemesis or neurologic symptoms [4]. The diagnostic tool of choice is computerized tomography (CT) scan of the chest [1, 3]. Surgical procedures noted in the literature include esophageal ligation and decompression, stenting of the esophagus and direct intracardiac or transthoracic repair with or without cardiopulmonary bypass (CPB; [5]). In this paper, we report successful repair via a novel surgical approach used in three patients.

## CASE REPORT

**Figure 1:**
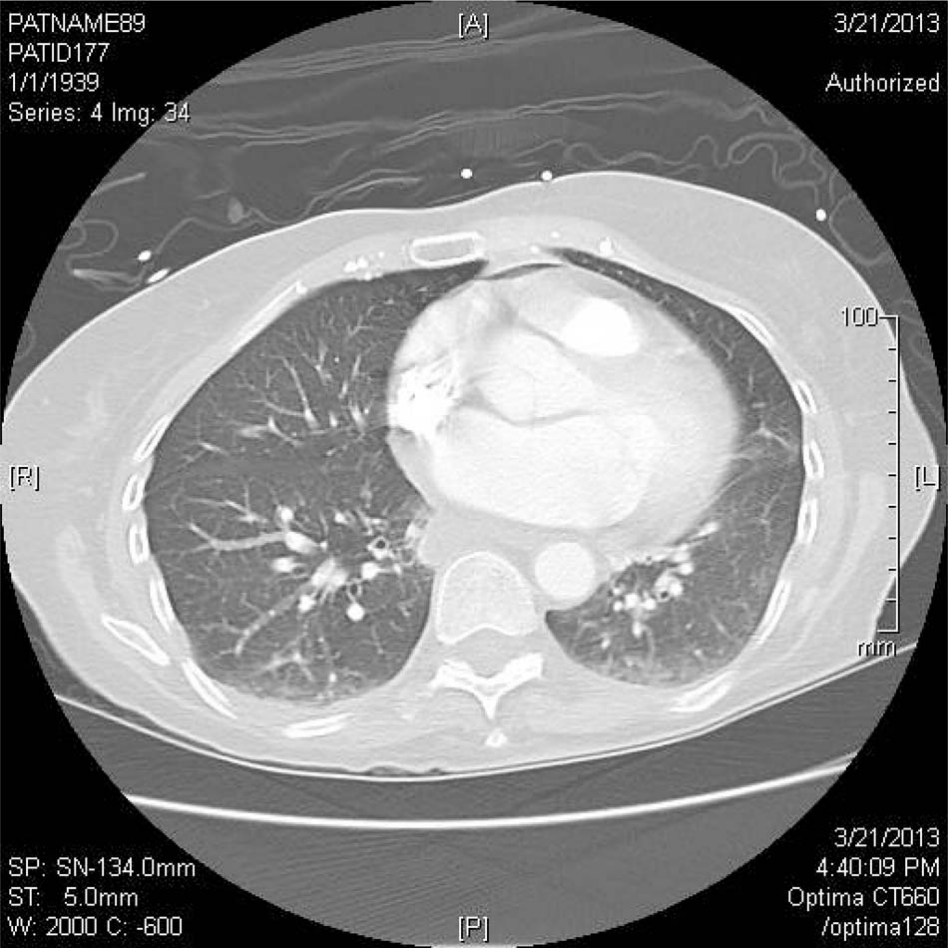
CT scan revealing pneumomediastinum.

Three patients have been treated by a single surgeon for left AEF. All presented with similar symptoms: chest pain, fever and dysphagia. The diagnosis was confirmed on CT scan of the chest (Fig. 1).

The patient undergoes rapid sequence intubation with a double lumen endotracheal tube. Once the tube is placed a bronchoscopy is performed to confirm position.

An esophagogastroduodenoscopy (EGD) is performed without insufflation. The scope is advanced into the stomach. A percutaneous gastrostomy tube (PEG) is placed using the pull technique. On withdrawal of the scope, the esophagus is inspected to determine the location of the fistula.

The patient is placed in the left lateral decubitus position and single lung ventilation is initiated. A right posterolateral muscle sparing thoracotomy is performed in the fifth intercostal space. At the time of the thoracotomy an intercostal muscle flap is harvested.

**Figure 2:**
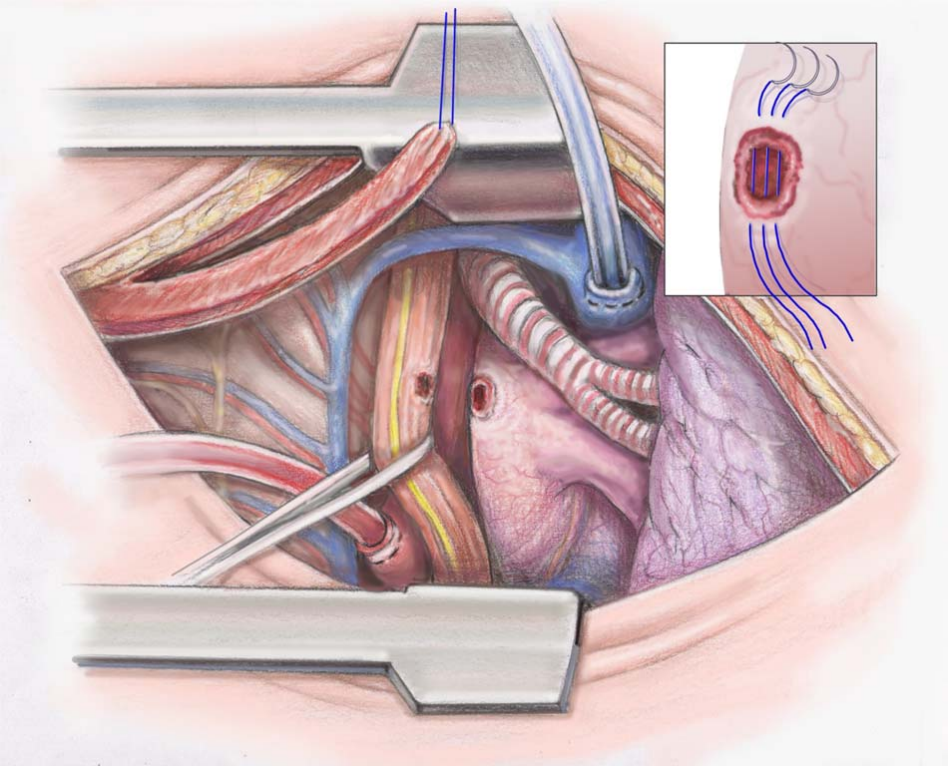
Artist illustration shows closure of atrial portion of fistula.

The descending aorta is exposed and 1 inch above the diaphragm a purse string suture is placed. The cannula is inserted and the suture secured. The pericardium is opened. If any purulent material is encountered, it is sent for culture. A 2–0 Ethibond purse string suture is placed on the lateral surface of the right atrium and cannulation is performed (Fig. 2).

Prior to placing the patient on CPB, sharp dissection of the esophagus is performed. The lung is retracted towards the mediastinum and the posterior mediastinal pleura is dissected. The esophagus is mobilized both proximal and distal to the fistula.

Once dissection of the esophagus is complete the patient is placed on CPB and fully heparinized. The fistula tract of the left atrium is opened and necrotic tissue debrided. It is essential that during debridement the surgeon skives toward the esophagus. A primary repair of the left atrium is then completed in an interrupted fashion using 4–0 prolene (Fig. 2). The heart is filled slowly and weaned off CPB.

**Figure 3:**
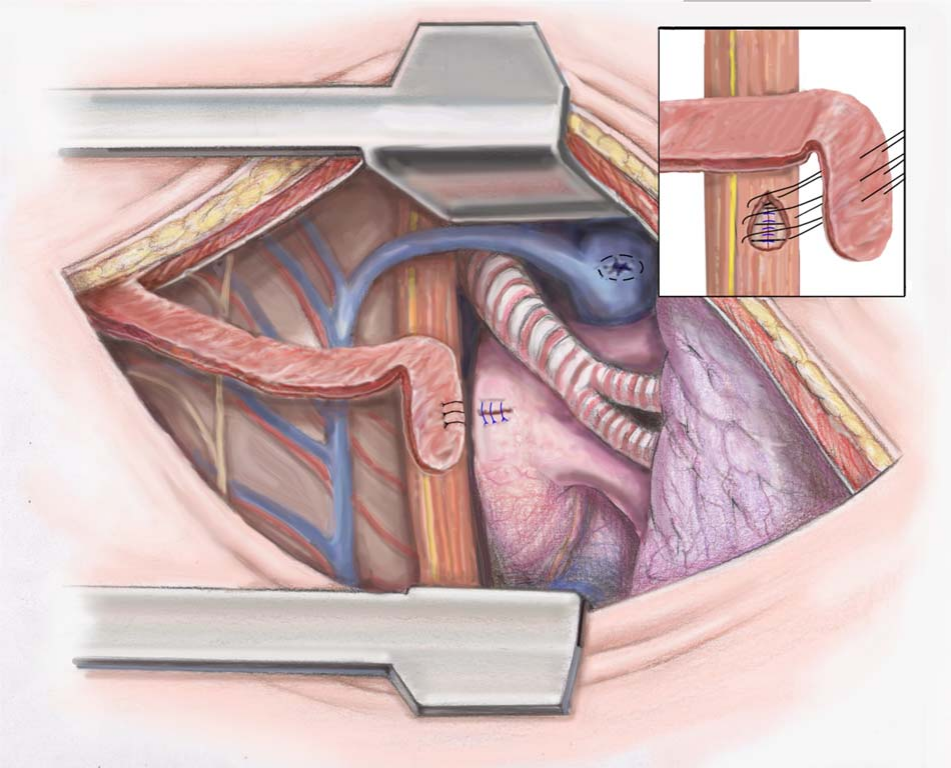
Artist illustration shows closure of esophageal portion of fistula and intercostal muscle flap placement.

Attention is then turned to the esophageal repair. The necrotic tissue is further debrided. The esophagus is closed in two layers. The inner serosal layer is repaired using a running 4–0 PDS suture. The outer muscular layer is repaired with interrupted 3–0 silk (Fig. 3).

To separate the repairs the intercostal muscle flap previously harvested is positioned. If the blood supply of the intercostal muscle is damaged, we then use the latissimus muscle, which was spared in the thoracotomy. The muscle flap is sutured in place (Fig. 3). The chest is drained widely using chest tubes and closed in a standard fashion.

## DISCUSSION

AEF is a life-threatening complication of ablation for treatment of atrial fibrillation [4]. This complication has a variable presentation, which leads to delayed recognition and high mortality rates [6]. The incidence of this complication is 0.03–1.5%, but the mortality rate is 40–80% [3–7].

The fistula formation is due to intraluminal thermal injury to the esophagus secondary to close approximation to the left atrium [3, 4, 8]. The thermal injury occurs when ablation is performed at the posterior portion of the left atrium. Injury can lead to ulceration of the esophagus [6]. Ulceration is likely the first step in the pathogenesis and other mechanisms including injury to esophageal blood supply, fat necrosis, reflux disease and/or injury to the vagus nerve may lead to development of the fistula [6].

Procedural risk factors include general anesthesia and esophageal temperatures > 41°C [3, 7]. Anatomical risk factors include short atrial to esophageal distance, small left atrium and absence of a fat layer between the left atrium and esophagus [1, 3]. Patient factors include advanced age, congestive heart failure, males and low body mass index [1, 2, 7].

The clinical presentation is variable. The most common triad includes fever, neurologic deficit and hematemesis [1]. Additional symptoms include dysphagia, nausea, heartburn, pleural effusion and chest pain [1, 3, 4]. Patients typically present 20 days post ablation in our case series the presentation ranged from 2 days to 2 weeks [1].

CT chest is the confirmatory test of choice [1, 3, 6, 8]. Findings include pericardial effusion, intravascular air, communication between the atrium and the pericardium or esophagus, and extensive systemic emboli [3]. Pneumomediastinum is a strong indicator of esophageal injury [1]. For patients who have neurologic symptoms a CT head is recommended [1].

If this complication is untreated, it is fatal [3, 5]. There are two predominant treatment strategies: esophageal stenting and surgical repair [3]. Previous articles have described esophageal ligation and decompression and direct intracardiac or transthoracic repair with or without CPB [5].

Our surgical intervention allows for one incision and multiple options for muscle flap by sparing the latissimus dorsi muscle. At the beginning of our procedure, we perform an EGD without insufflation not only to place a PEG tube for nutrition but to visualize the fistula. It is performed without insufflation to decrease the risk of embolism to the brain. We approach the right chest as it allows for easier mobilization of the esophagus and it allows access to the right atrium for central cannulation. Our novel approach has been successful in the three patients above.

## References

[1] Chavez P , Messerli F, Dominguez AC, Aziz EF, Sichrovsky T, Garcia D, et al. Atrioesophageal fistula following ablation procedures for atrial fibrillation: systematic review of case reports. Open Heart 2015;2:e000257. 26380098 10.1136/openhrt-2015-000257 PMC4567782

[2] Dagres N , Hindricks G, Kottkamp H, Sommer P, Gaspar T, Bode K, et al. Complications of atrial fibrillation ablation in a high-volume center in 1,000 procedures: still cause for concern? J Cardiovasc Electrophys. 2009;20:1014–9. 10.1111/j.1540-8167.2009.01493.x 19490383

[3] Smith GA , Kleiman AM. A rare iatrogenic atrial-esophageal fistula and anesthetic considerations for primary surgical repair. J Cardiothorac Vasc Anesthesia. 2017;31:2156–60. 10.1053/j.jvca.2017.04.016 28911899

[4] Haggerty KA , George TJ, Arnaoutakis GJ, Barreiro CJ, Shah AS, Sussman MS. Successful repair of an atrioesophageal fistula after catheter ablation for atrial fibrillation. Ann Thorac Surg. 2012;93:313–5. 22186458 10.1016/j.athoracsur.2011.05.050

[5] Hartman AR , Glassman L, Katz S, Chinitz L, Ross W. Surgical repair of a left atrial-esophageal fistula after radiofrequency catheter ablation for atrial fibrillation. Ann Thorac Surg 2012;94:e91–3. 23006721 10.1016/j.athoracsur.2012.04.052

[6] Singh SM , D’Avila A, Singh SK, Stelzer P, Saad EB, Skanes A, et al. Clinical outcomes after repair of left atrial esophageal fistulas occurring after atrial fibrillation ablation procedures. Heart Rhythm. 2013;10:1591–7. 23954269 10.1016/j.hrthm.2013.08.012

[7] Vasconcelos JTMD , Filho SDSG, Atié J, Maciel W, Souza OFD, Saad EB, et al. Atrial-oesophageal fistula following percutaneous radiofrequency catheter ablation of atrial fibrillation: the risk still persists. Europace. 2017;19:250–8. 28175286 10.1093/europace/euw284

[8] Gray WH , Fleischman F, Cunningham MJ, Kim AW, Baker CJ, Starnes VA, et al. Optimal approach for repair of left atrial-esophageal fistula complicating radiofrequency ablation. Ann Thoracic Surg 2018;105:e229–31. 10.1016/j.athoracsur.2017.12.026 29410186

